# Curling Cuticles of the Great Toenails: A Case Report of Eponychogryphosis

**DOI:** 10.7759/cureus.3959

**Published:** 2019-01-25

**Authors:** Philip R Cohen

**Affiliations:** 1 Dermatology, San Diego Family Dermatology, San Diego, USA

**Keywords:** curl, curling, cuticle, eponychium, eponychogryphosis, fold, great, onychogryphosis, nail, toe

## Abstract

The cuticle, also referred to as the eponychium, creates a seal between the proximal nail fold and the nail plate. It is derived from both the ventral and dorsal portions of the proximal nail fold. In addition to its principle function as a barrier preventing allergens, irritants and pathogens from entering the nail cul-de-sac, the cuticle can play a role as a model for evaluating the etiology and management of diseases that affect capillary microcirculation, provide a source of solid tissue for genetic disorder studies, and aid in the evaluation of patients in whom the diagnoses of either systemic scleroderma or dermatomyositis is being entertained. Curling cuticle is a distinctive and unique occurrence. The clinical features of a man with curling cuticles on the lateral portion of both great toes is described. Although a deficiency in personal hygiene may partially account for the clinical finding, the pathogenesis of this observation remains to be established. The term ‘eponychogryphosis’ is proposed to describe the alteration of the patient’s cuticles.

## Introduction

The cuticle, also known as the eponychium, is an extension of the stratum corneum from the proximal nail fold [1-3]. It forms a seal that prevents allergens, irritants, and pathogens from entering the potential space between the distal skin of the digit and the nail plate [4-5]. An elderly man with curling cuticles on the lateral aspects of both of his great toenails is reported and a descriptive term for this condition is proposed: eponychogryphosis.

## Case presentation

An 83-year-old man presented for a total body skin check. He had a prior history of 16 nonmelanoma skin cancers, actinic keratoses, seborrheic dermatitis, stasis dermatitis, and tinea pedis. In addition to sleep apnea (which was treated with two liters of oxygen via continuous positive airway pressure each night), his medical history was significant for thyroid (hypothyroidism), cardiac (congestive heart failure, coronary atherosclerosis, hyperlipidemia, hypertension, paroxysmal atrial fibrillation, and sick sinus syndrome) and renal (chronic kidney disease) conditions. His daily medications included amlodipine, carvedilol, febuxostat, ferrous sulfate, finasteride, folic acid, furosemide, levoxyl, pravastatin, tamsulosin, and warfarin.

**Figure 1:**
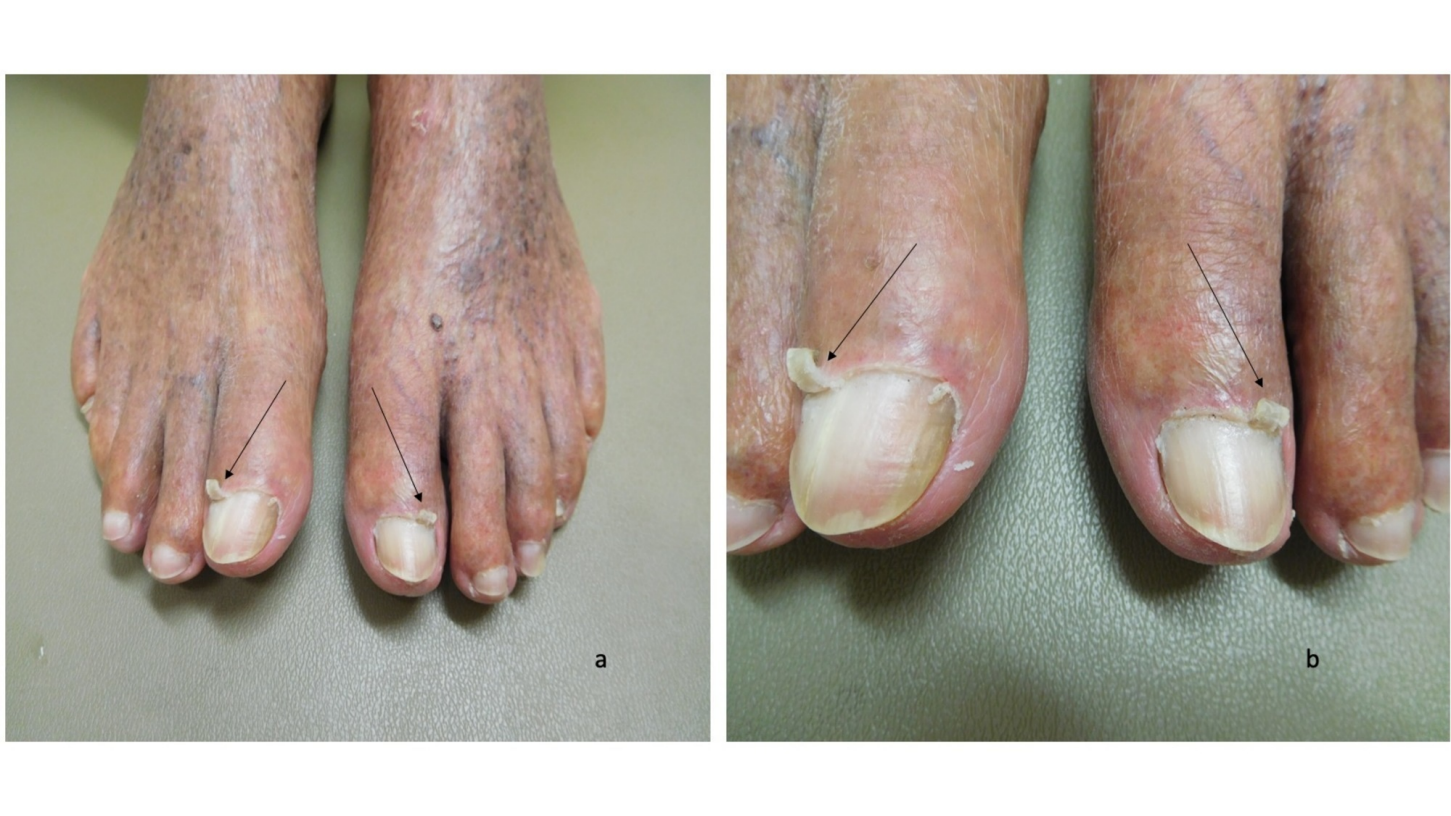
Curling cuticles (eponychogryphosis) of the great toes: both feet Distant (a) and closer (b) views of the dorsal great toes show elongation of the lateral aspect of the cuticle (black arrows).

**Figure 2:**
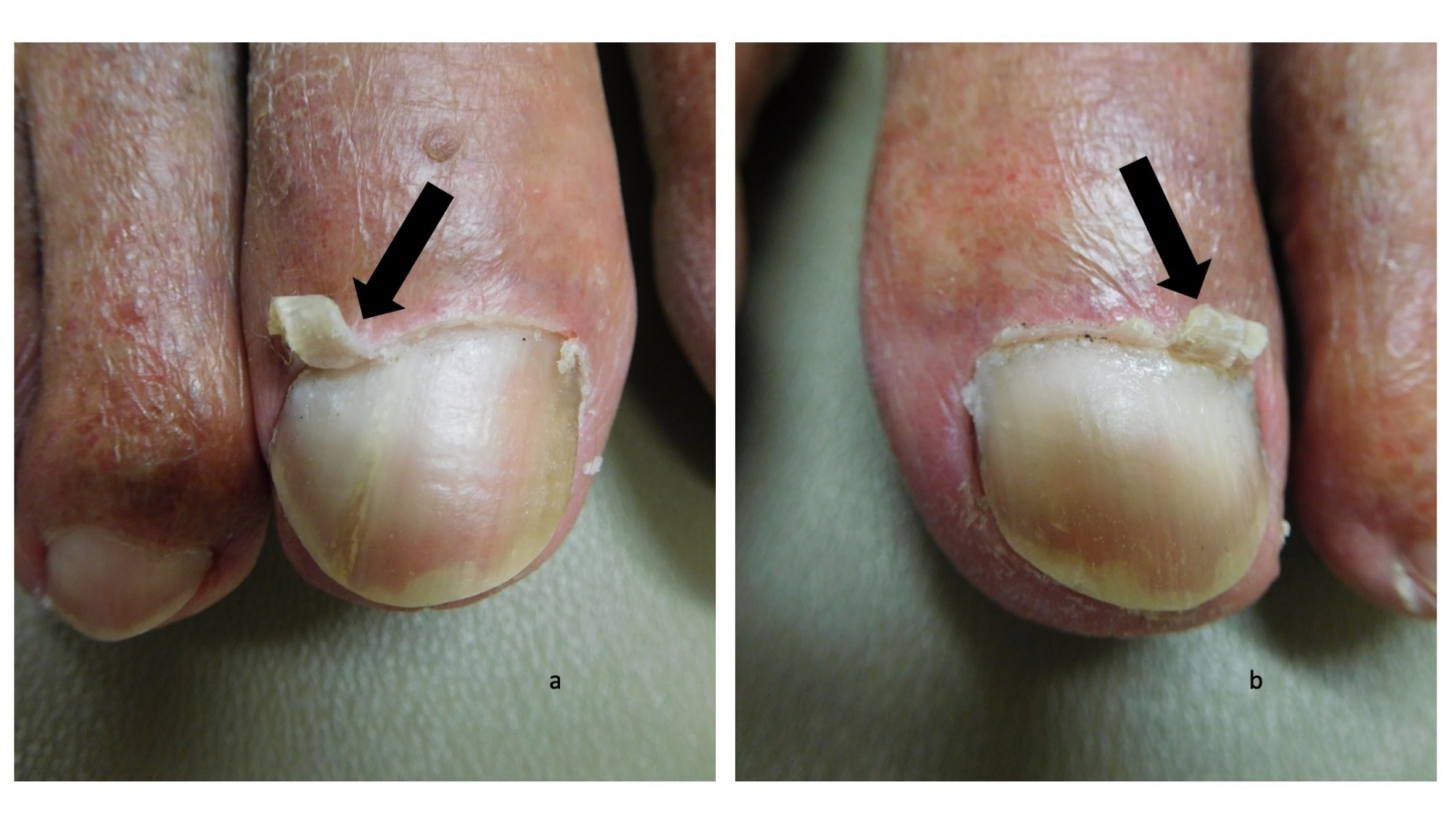
Curling cuticles (eponychogryphosis) of the great toes: right and left great toes The right great toe (a) and left great toe (b) shows curling of the lateral aspect of the cuticle (black arrows).

**Figure 3:**
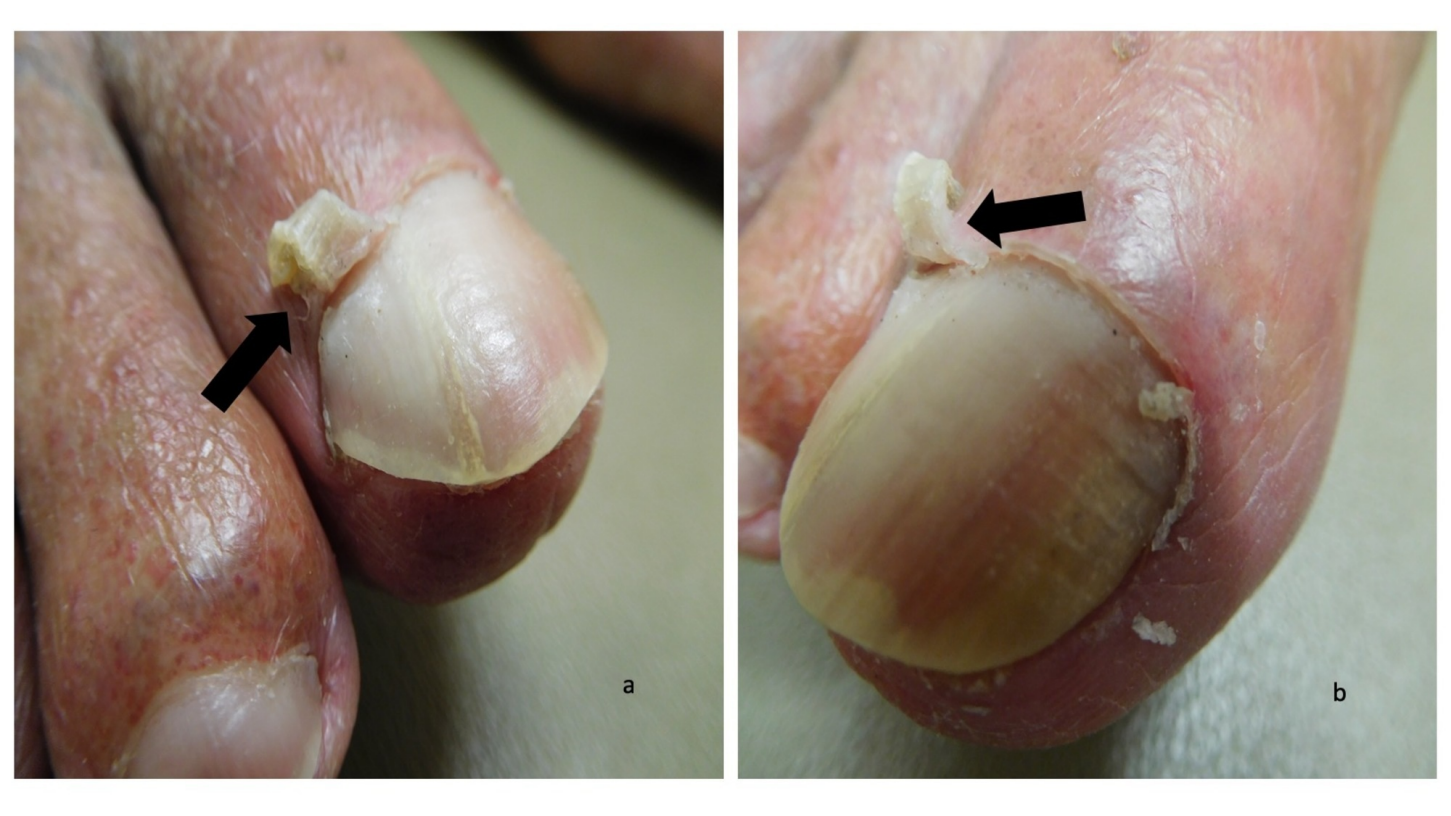
Curling cuticles (eponychogryphosis) of the great toes: right great toe Curling of the cuticle (black arrows) is shown on the lateral (a) and medial (b) views of the right great toe.

**Figure 4:**
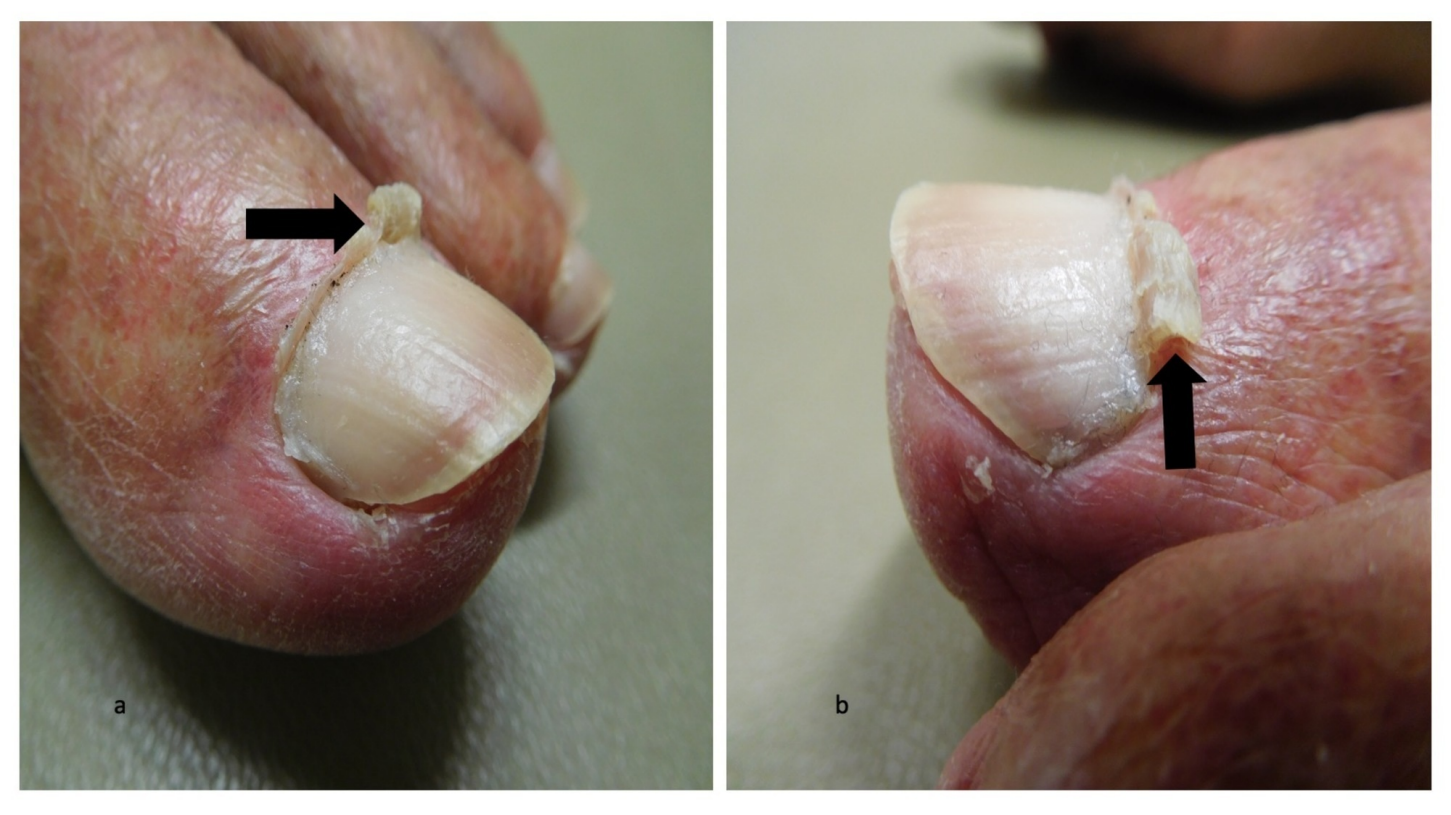
Curling cuticles (eponychogryphosis) of the great toes: left great toe Curling of the cuticle (black arrows) is shown on the medial (a) and lateral (b) views of the left great toe.

Cutaneous examination demonstrated keratotic plaques on the scalp, face, and arms; the actinic keratoses were treated with liquid nitrogen cryotherapy. Both great toes had an elongation of the lateral aspect of the cuticle as seen in Figure 1; the remainder of the cuticle was normal in appearance (Figure 2). Lateral and medial views of the right and left great toes revealed that there was curling of the cuticles (Figures 3-4).

A descriptive diagnosis, based on the clinical presentation, of curling cuticles was established. The patient had not been aware of his elongated and curly cuticles; indeed, additional history also revealed that he was not able to provide adequate hygiene to his toes since he could not reach them. Treatment was subsequently performed during a pedicure; the toes were soaked in water for five minutes and the curled cuticles were carefully cut at their base so that the cuticle was smooth and intact.

## Discussion

The nail unit consists not only of the nail plate but also the nail matrix, the nail bed, the hyponychium, and the nail folds. There are two lateral nail folds and a proximal nail fold. The proximal nail fold has a dorsal surface (which is continuous with the skin of the distal digit) and a ventral surface (which consists of two parts: a proximal portion that forms the proximal or apical matrix and the remaining distal portion that forms the eponychium). The cuticle is the stratum corneum that extends from both the dorsal and ventral (eponychium) surface of the proximal nail fold; however, eponychium and cuticle are considered to be synonyms since many researchers interchangeably use the terms in the medical literature. The cuticle morphologically appears as a horny layer of desquamating tissue and provides a seal between the proximal nail fold and the nail plate to protect the ungual cul-de-sac [1-3].

The keratin composition of the cuticle is unique. Immunohistochemical analysis of epithelial and hair keratins has demonstrated that the ventral aspect of the proximal nail fold consists of two distinctive epithelial tissue compartments. The proximal nail matrix (which forms the superficial portion of the nail plate) exhibits a mixed pattern of epithelial and hair keratin expression. In contrast, the cuticle only expresses epithelial keratins [1].

The function of the cuticle is to be a protective barrier against the entry of infectious organisms, external allergens, and irritating substances into the space between the proximal nail and the nail plate. Indeed, an intact cuticle provides a water-proof seal. However, there are several situations that can result in damage to the cuticles: contact with chemicals used during the application of artificial nails, cuticle removal during manicures and pedicures, finger sucking, hand washing excessively, nail biting, and nail picking [4-5].

Acute and chronic paronychia may occur when the function of the cuticle is disrupted. Direct or indirect minor trauma to either the cuticle or the nail fold can result in an acute paronychia. Often there is an associated bacterial (such as Staphylococcus aureus) or viral (such as herpes simplex virus) pathogen that invades the periungual soft tissue or nail cul-de-sac and causes abscess formation with swelling, tenderness, and erythema. Systemic antimicrobial or antiviral systemic therapy if frequently necessary [4].

Improper treatment of acute paronychia or persistent injury to the cuticle, such that its barrier function is disrupted, may result in inflammation and chronic paronychia. In this setting, secondary colonization by fungal (such as Candida species) or bacterial organisms may also occur. Since the primary problem of chronic paronychia is the inflammatory reaction, topical application of corticosteroids (with or without systemic or topical antifungal or antibacterial agents) should be initiated [3-5].

Although the cuticle’s principle function is to serve as a sealant, investigators have also utilized the cuticle for evaluating capillary microcirculation [6], for performing genetic studies [7], and for aiding in the diagnosis of autoimmune connective tissue diseases [8-9]. Researchers developed a technique to evaluate the microcirculation in humans by measuring the pressure of the capillaries in the cuticles of the fourth finger. The method they developed using cuticle capillaries will enable them to achieve further insight into the pathogenesis and therapeutic intervention of diseases such as diabetes mellitus, peripheral vascular disease, and vasospastic disorders that affect the microcirculation [6].

The cuticle is an easily obtainable source of tissue for evaluation in genetic studies. Glucose-6-phosphate dehydrogenase deficiency is an inborn error in metabolism; there is variability of enzyme activity in patients with the x-linked Mediterranean variant of this condition. Investigators were able to demonstrate that the enzyme assay of not only the hair, but also the cuticles could accurately identify individuals with the Mediterranean type of glucose-6-phosphate dehydrogenase deficiency [7].

Nail fold capillaroscopy of the cuticles can be useful for aiding in the diagnosis of certain diseases such as systemic sclerosis and dermatomyositis. Both of these conditions (during the acute phase of disease activity) have characteristic nail fold findings. These include dilated capillaries, cuticular (eponychial) hemosiderin-containing deposits (CEHD), and microhemorrhages that can be readily visualized using a ten-fold magnification dermoscopy [8-9].

The observation of an individual with curling cuticles, to the best of my knowledge, has not previously been described. This morphologic development is indeed unique. In part, the elongated cuticles appear to be secondary to a deficiency in personal hygiene. The reported man was unable to provide careful attention to the grooming of the cuticles on his great toenails; this resulted not only because he had difficulty reaching his toes, but also since he was unaware of the elongated cuticles. However, the pathogenesis for the distinctive hyperplastic growth of that portion of his cuticles remains to be determined.

Nails that clinically present with a ram’s horn-like deformity are referred to as onychogryphosis; ‘onycho’ is a prefix for ‘pertaining to nails’ and ‘gryphosis’ means ‘abnormal curvature.’ The reported patient had abnormal curvature of his eponychium. Therefore, the term ‘eponychogryphosis’ is proposed to appropriately describe the alteration of his cuticles.

## Conclusions

The cuticle—the stratum corneum derived from both the ventral (distal eponychium) and dorsal portions of the proximal nail fold—creates a seal between the proximal nail fold and the nail plate so that allergens, irritants, and pathogens do not enter the nail cul-de-sac; however, acute or chronic paronychia may develop when the integrity of the cuticle is compromised. In addition, the cuticle can not only serve as a model for evaluating the etiology and management of diseases that affect capillary microcirculation, but also as a source of solid tissue to study genetic disorders. Also, nail fold capillaroscopy of the cuticle may be useful in the assessment of patients in whom the diagnoses of either systemic scleroderma or dermatomyositis is being considered. The clinical features of a man with curling cuticles on the lateral portion of both great toes is described. The pathogenesis of this unique morphologic development remains to be established. Deficiency in personal hygiene, in part, may account for the occurrence, but would not account for the selective distribution of the cuticle growth. To appropriately describe the alteration of the reported patient’s cuticles, the term ‘eponychogryphosis’ is proposed.
